# Supplementary material for: The Impact of a Commercial Biostimulant on the Grape Mycobiota of Vitis vinifera cv. Barbera
Source: Microorganisms. 2023 Jul 25;11(8):1873. doi: 10.3390/microorganisms11081873 (PMC10457965; doi:10.3390/microorganisms11081873)
Supplement: Supplementary file 1 [file microorganisms-11-01873-s001.zip › microorganisms-2457044-supplementary.pdf]

Table S1. Alpha biodiversity data. Recorded data of the alpha biodiversity in terms of number of observed species, Shannon's and Simpson's index, as output from MicrobiomeAnalyst. In bold type are highlighted the median values.

Thesis	sample	Species Observed (p = 0,049894)	Shannon's Index (p = 0,73345)	Simpson's Index (p = 0,56553)
Control (C)	R3P18C	65	1,14	0,558
	R5P8C	**79**	**1,41**	0,637
	R6P11C	93	1,67	0,646
	R7P13C	84	1,45	**0,606**
	R8P1C	63	1,05	0,503
Bion (B)	R9P3B	**83**	1,11	0,455
	R9P11B	86	1,59	**0,618**
	R10P6B	94	1,30	0,543
	R10P16B	69	1,61	0,699
	R11P7B	75	**1,53**	0,713
Water (W)	R13P6W	57	1,41	0,696
	R13P13W	51	**1,44**	**0,667**
	R13P23W	**60**	1,49	0,688
	R14P9W	83	1,51	0,604
	R14P17W	62	1,39	0,586

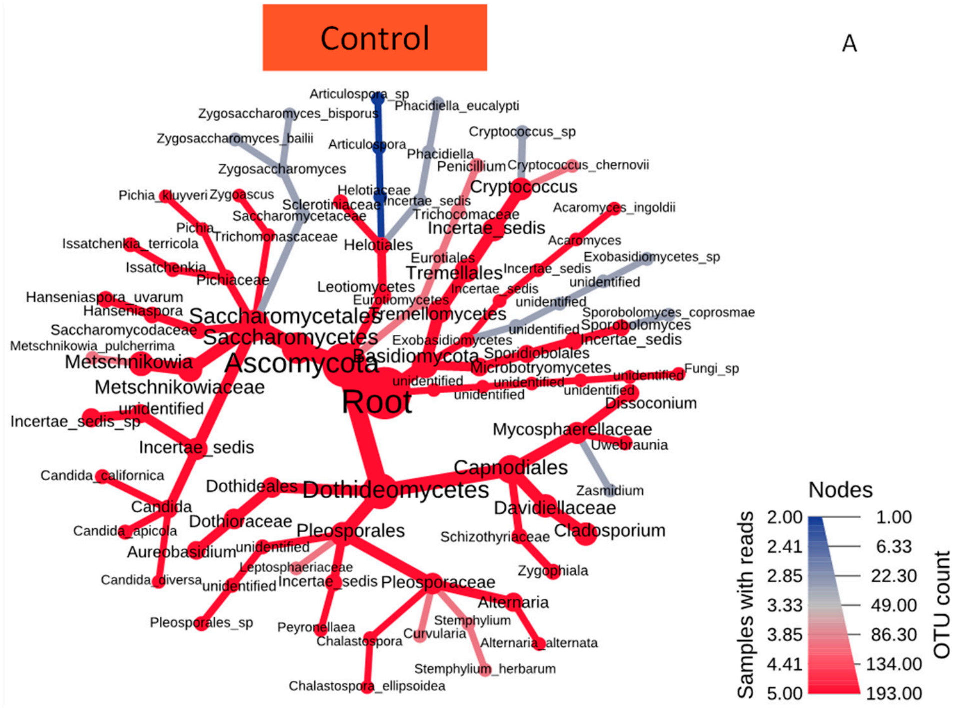
Control
A
Articulospora_sp
Zygosaccharomyces_bisporus
Phacidiella_eucalypti
Zygosaccharomyces_bailii
Cryptococcus_sp
Articulospora
Phacidiella
Zygosaccharomyces
Penicillium
Cryptococcus_chernovii
Pichia_kluyveri
Zygoascus
Helotiaceae
Cryptococcus
Sclerotiniaceae
Incertae_sedis
Acaromyces_ingoldii
Saccharomycetaceae
Trichocomaceae
Pichia
Incertae_sedis
Issatchenkia_terricola
Trichomonascaceae
Acaromyces
Helotiales
Eurotiales
Exobasidiomycetes_sp
Issatchenkia
Tremellales
Incertae_sedis
Pichiaceae
unidentified
Leotiomycetes
Incertae_sedis
Hanseniaspora_uvarum
Eurotiomycetes
unidentified
Hanseniaspora
Saccharomycetales
Sporobolomyces_coprosmae
Tremellomycetes
unidentified
Sporobolomyces
Saccharomycodaceae
Saccharomycetes
Incertae_sedis
Metschnikowia_pulcherrima
Exobasidiomycetes
Basidiomycota
Sporidiobolales
Metschnikowia
Ascomycota
Microbotryomycetes
unidentified
Fungi_sp
unidentified
unidentified
Metschnikowiaceae
unidentified
unidentified
Root
unidentified
Dissoconium
Incertae_sedis_sp
Mycosphaerellaceae
Incertae_sedis
Uwebraunia
Candida_californica
Capnodiales
Dothideales
Dothideomycetes
Zasmidium
Candida
Davidiellaceae
Dothioraceae
Candida_apicola
Pleosporales
Cladosporium
Schizothyriaceae
Aureobasidium
unidentified
Leptosphaeriaceae
Zygophiala
Candida_diversa
unidentified
Incertae_sedis
Pleosporaceae
Alternaria
Pleosporales_sp
Stemphylium
Peyronellaea
Curvularia
Chalastospora
Alternaria_alternata
Stemphylium_herbarum
Chalastospora_ellipsoidea
Nodes
Samples with reads
2.00
2.41
2.85
3.33
3.85
4.41
5.00
OTU count
1.00
6.33
22.30
49.00
86.30
134.00
193.00

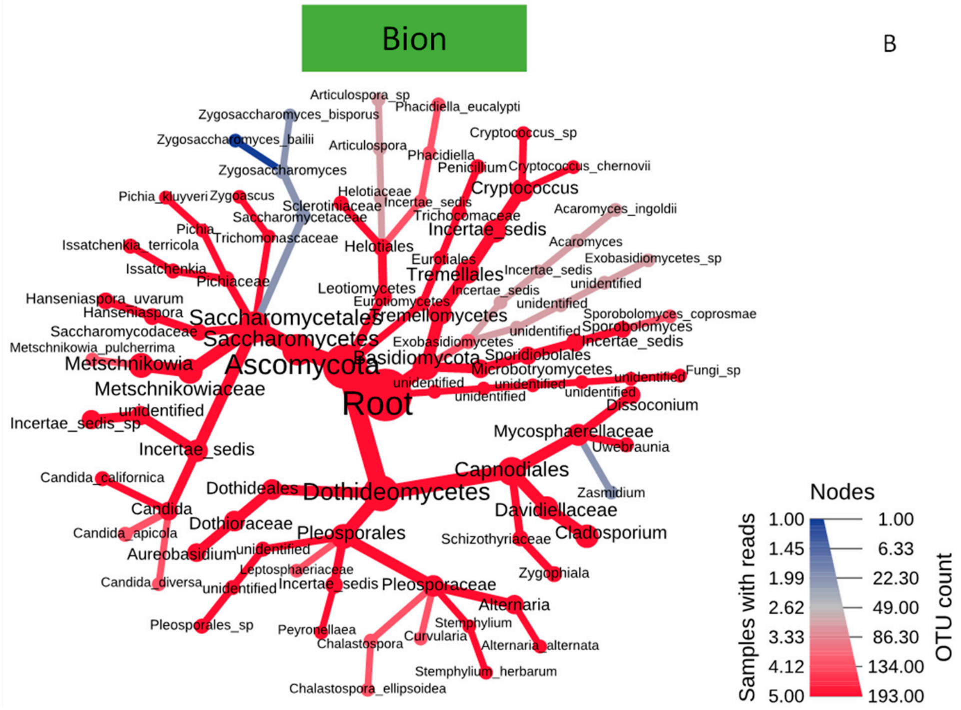
Bion
B
Articulospora_sp
Zygosaccharomyces_bisporus
Phacidiella_eucalypti
Zygosaccharomyces_bailii
Cryptococcus_sp
Articulospora
Phacidiella
Zygosaccharomyces
Penicillium
Cryptococcus_chernovii
Pichia_kluyveri
Zygoascus
Helotiaceae
Cryptococcus
Sclerotiniaceae
Incertae_sedis
Acaromyces_ingoldii
Saccharomycetaceae
Trichocomaceae
Pichia
Incertae_sedis
Issatchenkia_terricola
Trichomonascaceae
Acaromyces
Helotiales
Eurotiales
Exobasidiomycetes_sp
Issatchenkia
Tremellales
Incertae_sedis
Pichiaceae
unidentified
Leotiomycetes
Incertae_sedis
Hanseniaspora_uvarum
Eurotiomycetes
unidentified
Hanseniaspora
Saccharomycetales
Sporobolomyces_coprosmae
Tremellomycetes
unidentified
Sporobolomyces
Saccharomycodaceae
Saccharomycetes
Incertae_sedis
Metschnikowia_pulcherrima
Exobasidiomycetes
Basidiomycota
Sporidiobolales
Metschnikowia
Ascomycota
Microbotryomycetes
unidentified
Fungi_sp
unidentified
unidentified
Metschnikowiaceae
unidentified
unidentified
Root
unidentified
Dissoconium
Incertae_sedis_sp
Mycosphaerellaceae
Incertae_sedis
Uwebraunia
Candida_californica
Capnodiales
Dothideales
Dothideomycetes
Zasmidium
Candida
Davidiellaceae
Dothioraceae
Candida_apicola
Pleosporales
Cladosporium
Schizothyriaceae
Aureobasidium
unidentified
Leptosphaeriaceae
Zygophiala
Candida_diversa
unidentified
Incertae_sedis
Pleosporaceae
Alternaria
Pleosporales_sp
Stemphylium
Peyronellaea
Curvularia
Chalastospora
Alternaria_alternata
Stemphylium_herbarum
Chalastospora_ellipsoidea
Nodes
Samples with reads
1.00
1.45
1.99
2.62
3.33
4.12
5.00
OTU count
1.00
6.33
22.30
49.00
86.30
134.00
193.00

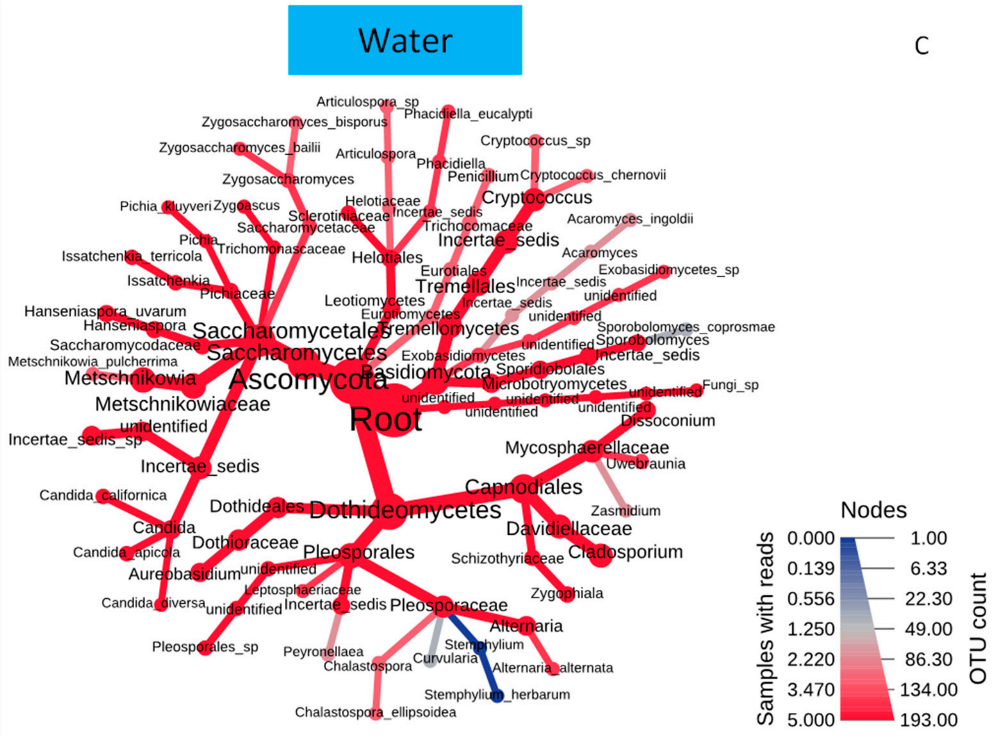

Figure S1. Heat tree chart. comparison of abundance at species level with heat tree method in each treatment.

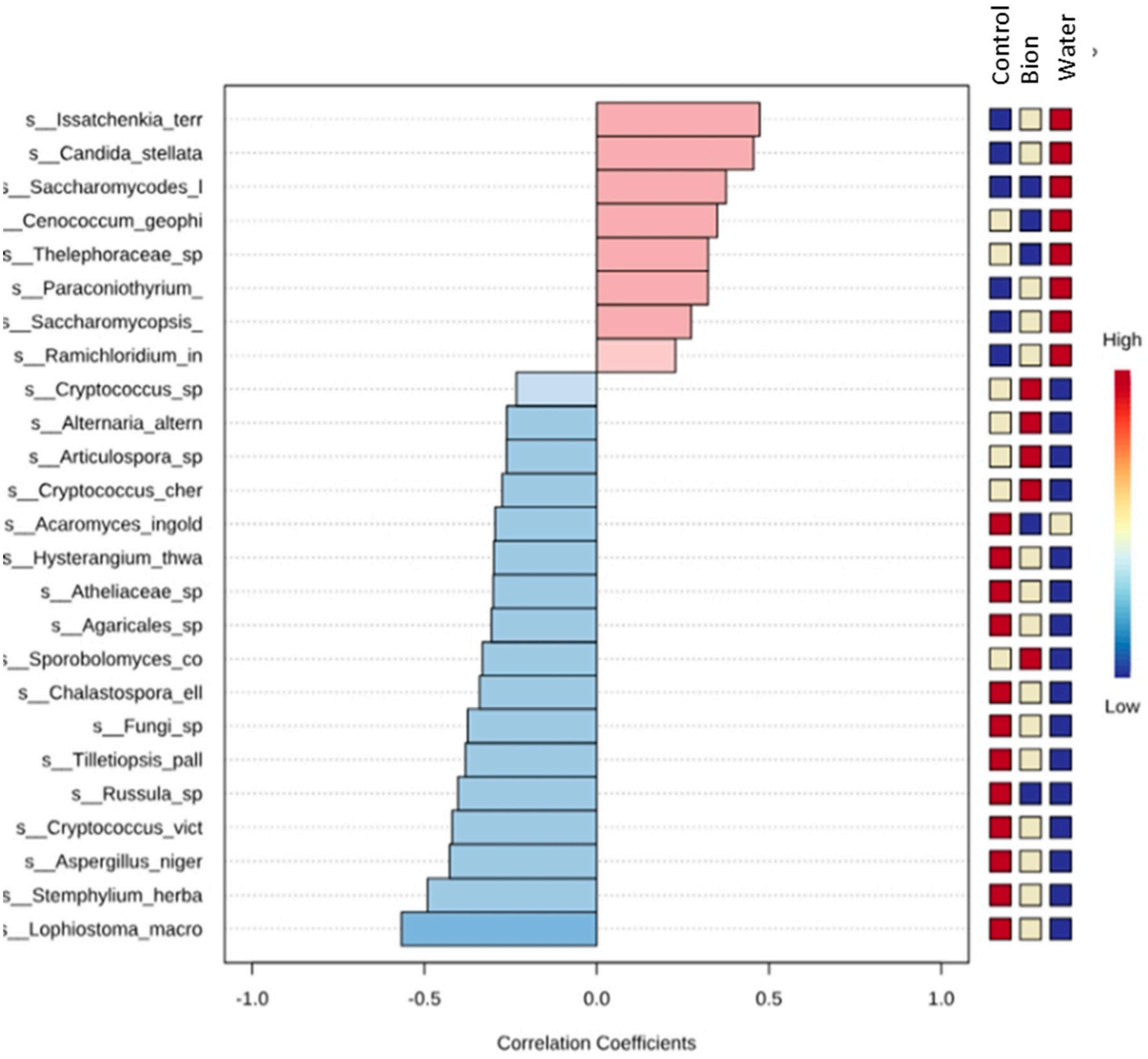

Figure S2. Correlation coefficients. Top 25 OTUs have been correlated with the three treatments.

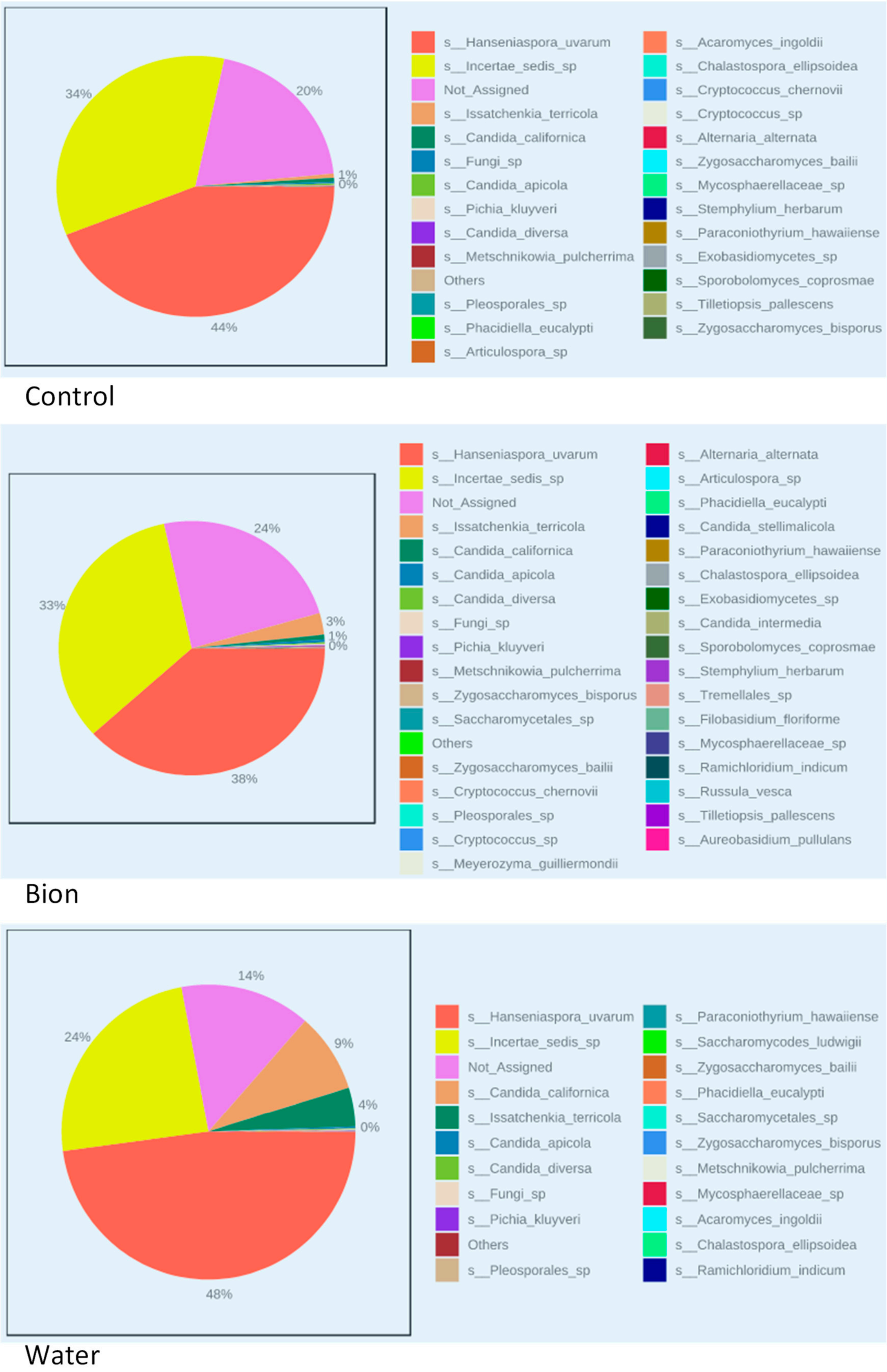

Figure S3. Pie-Chart of relative abundance at species level. Relative abundance in percentage of the species detected in each thesis.
